# Contrast-Enhancing Lesion Segmentation in Multiple Sclerosis: A Deep Learning Approach Validated in a Multicentric Cohort

**DOI:** 10.3390/bioengineering11080858

**Published:** 2024-08-22

**Authors:** Martina Greselin, Po-Jui Lu, Lester Melie-Garcia, Mario Ocampo-Pineda, Riccardo Galbusera, Alessandro Cagol, Matthias Weigel, Nina de Oliveira Siebenborn, Esther Ruberte, Pascal Benkert, Stefanie Müller, Sebastian Finkener, Jochen Vehoff, Giulio Disanto, Oliver Findling, Andrew Chan, Anke Salmen, Caroline Pot, Claire Bridel, Chiara Zecca, Tobias Derfuss, Johanna M. Lieb, Michael Diepers, Franca Wagner, Maria I. Vargas, Renaud Du Pasquier, Patrice H. Lalive, Emanuele Pravatà, Johannes Weber, Claudio Gobbi, David Leppert, Olaf Chan-Hi Kim, Philippe C. Cattin, Robert Hoepner, Patrick Roth, Ludwig Kappos, Jens Kuhle, Cristina Granziera

**Affiliations:** 1 Translational Imaging in Neurology (ThINk) Basel, Department of Biomedical Engineering, Faculty of Medicine, University Hospital Basel, University of Basel, 4123 Basel, Switzerland; martina.greselin@unibas.ch (M.G.); riccardo.galbusera@usb.ch (R.G.); alessandro.cagol@unibas.ch (A.C.); esther.ruberte@unibas.ch (E.R.);; 2 Department of Neurology, University Hospital Basel, 4031 Basel, Switzerland; jens.kuhle@usb.ch; 3 Research Center for Clinical Neuroimmunology and Neuroscience Basel (RC2NB), University Hospital Basel, University of Basel, 4031 Basel, Switzerland; 4 Department of Health Sciences, University of Genova, 16132 Genova, Italy; 5 Division of Radiological Physics, Department of Radiology, University Hospital Basel, 4031 Basel, Switzerland; 6 Medical Image Analysis Center (MIAC), 4051 Basel, Switzerland; 7 Clinical Trial Unit, Department of Clinical Research, University Hospital Basel, University of Basel, 4031 Basel, Switzerland; 8 Department of Neurology, Cantonal Hospital St. Gallen, 9000 St. Gallen, Switzerland; 9 Department of Neurology, Cantonal Hospital Aarau, 5001 Aarau, Switzerland; 10 Neurology Department, Neurocenter of Southern Switzerland, 6900 Lugano, Switzerland; 11 Department of Neurology, Inselspital, Bern University Hospital, University of Bern, 3010 Bern, Switzerland; 12 Department of Neurology, St. Josef-Hospital, Ruhr-University Bochum, 44791 Bochum, Germany; 13 Service of Neurology, Department of Clinical Neurosciences, Lausanne University Hospital (CHUV), University of Lausanne, 1005 Lausanne, Switzerland; 14 Division of Neurology, Department of Clinical Neurosciences, Faculty of Medicine, Geneva University Hospitals, 1205 Geneva, Switzerland; 15 Faculty of biomedical Sciences, Università della Svizzera Italiana, 6962 Lugano, Switzerland; 16 Division of Diagnostic and Interventional Neuroradiology, Clinic for Radiology and Nuclear Medicine, University Hospital Basel, University of Basel, 4031 Basel, Switzerland; johanna.lieb@usb.ch; 17 Department of Radiology, Cantonal Hospital Aarau, 5001 Aarau, Switzerland; 18 Department of Diagnostic and Interventional Neuroradiology, Inselspital, Bern University Hospital, University of Bern, 3010 Bern, Switzerland; 19 Department of Radiology, Faculty of Medicine, Geneva University Hospital, 1205 Geneva, Switzerland; 20 Department of Neuroradiology, Neurocenter of Southern Switzerland, 6900 Lugano, Switzerland; 21 Department of Radiology, Cantonal Hospital St. Gallen, 9000 St. Gallen, Switzerland; 22 Center for medical Image Analysis & Navigation, Department of Biomedical Engineering, University of Basel, 4123 Allschwil, Switzerland; philippe.cattin@unibas.ch; 23 Department of Neurology, University Hospital of Zurich, University of Zurich, 8091 Zurich, Switzerland

**Keywords:** deep learning, multiple sclerosis, automatic segmentation, gadolinium contrast-enhancing lesions

## Abstract

The detection of contrast-enhancing lesions (CELs) is fundamental for the diagnosis and monitoring of patients with multiple sclerosis (MS). This task is time-consuming and suffers from high intra- and inter-rater variability in clinical practice. However, only a few studies proposed automatic approaches for CEL detection. This study aimed to develop a deep learning model that automatically detects and segments CELs in clinical Magnetic Resonance Imaging (MRI) scans. A 3D UNet-based network was trained with clinical MRI from the Swiss Multiple Sclerosis Cohort. The dataset comprised 372 scans from 280 MS patients: 162 showed at least one CEL, while 118 showed no CELs. The input dataset consisted of T1-weighted before and after gadolinium injection, and FLuid Attenuated Inversion Recovery images. The sampling strategy was based on a white matter lesion mask to confirm the existence of real contrast-enhancing lesions. To overcome the dataset imbalance, a weighted loss function was implemented. The Dice Score Coefficient and True Positive and False Positive Rates were 0.76, 0.93, and 0.02, respectively. Based on these results, the model developed in this study might well be considered for clinical decision support.

## 1. Introduction

Multiple sclerosis (MS) is a chronic autoimmune disorder affecting the central nervous system. It is characterized by the inflammatory infiltration of lymphocytes and macrophages; activation of microglia; and degeneration of myelin, axons, oligodendrocytes, and neurons [1,2]. In active lesions, the inflammatory process damages the blood–brain barrier, leading to the extravasation of gadolinium into the brain tissue, which is identifiable in post-contrast T1-weighted MR images [3]. Identifying these lesions is essential in the diagnosis of multiple sclerosis [4] and in evaluating the efficacy of treatments [5,6,7,8,9].

The detection and segmentation of these lesions are time-consuming and suffer from notable variability between different raters in various clinical settings [10]. Hence, the development of an automated tool for these tasks holds significant promise for clinical practice [11]. The automated tool should achieve an accurate identification/segmentation of CELs, thereby minimizing the time clinicians need to spend on reviewing results. Creating an automated tool for the detection of CELs involves various challenges, such as the small to only point-like enhancement size of CELs, their random location, and their heterogeneous shapes. While nodular shapes are more common, larger lesions may manifest as closed-ring CELs [12]. Additionally, lesions situated near the ventricles or cortex might exhibit open-ring enhancements in T1-weighted images with contrast agents [12].

Distinguishing CELs from physiological hyperintensities in post-contrast T1-weighted images (for example, veins) can be challenging [13]. To confirm the existence of real CELs and exclude False Positives, corresponding hyperintense areas in FLuid Attenuated Inversion Recovery (FLAIR) images must be identified. Indeed, CELs represent the acute inflammatory component within white matter lesions (WMLs) [12].

Deep learning architectures are extensively employed in various medical imaging applications due to their capability to learn and identify intricate patterns and features within images [14]. A Convolutional Neural Network (CNN) is a deep learning model that can be applied to process image data and is widely used for medical image segmentation [15].

Only a few studies have proposed automated tools for segmenting and detecting CELs [16,17,18]. Coronado et al. [17] developed a 3D CNN to automatically segment CELs, within a network trained to also segment white matter, gray matter, cerebrospinal fluid, and T2 lesions. Gaj et al. [16], developed a 2D U-Net which performs the initial segmentation followed by a postprocessing phase. It comprises a random forest classifier that integrates the 3D spatial information and performs the prediction. Furthermore, Krishnan et al. [18] developed a joint U-Net model to segment both T1 non-enhancing and CELs. These studies attempted to address the previously mentioned challenges in identifying CELs by expanding the input dataset with multiple contrast images [17] and implementing probability maps [16]. Moreover, these studies were performed in clinical trial datasets [17,18], excluding lesions with low volume to increase the performance [16,17,18].

Furthermore, Schlager et al. [19] proposed a 3D CNN to perform an automatic detection of CELs using a clinical routine dataset.

The transfer learning approach [20] presents a novel solution to address the limited dataset, which is due to the rarity of MS pathology, the sparse presence of CELs, and the difficulty in obtaining a manually segmented ground truth.

S.G. Wahlig et al. [21] employed a pretrained 3D Unet model on other pathologies to segment and detect MS lesions. The model was trained to identify enhancing brain lesions in the context of intracranial metastases. This model was then fine-tuned with the MS dataset, resulting in improved performance compared to a model trained solely on the MS dataset. This improvement is attributed to the statistical similarities between enhancing MS lesions and enhancing metastases.

Lan Huang et al. [22] utilized a 2.5D Fully Convolutional with Attention DenseNet (FCA-DenseNet) network to segment contrast-enhancing lesions (CELs) in contrast-enhancing T1-weighted images. The dataset comprises patients diagnosed with multiple sclerosis (MS) and neuromyelitis optica spectrum disorder (NMOSD). To address the challenges posed by the limited dataset and sparse lesions, they proposed a transfer learning approach. The 2.5D slicing strategy was employed to reduce model complexity, improve the training dataset, increase data features, and enhance segmentation performance.

This project aims to develop a deep learning model intended as a supportive tool for clinicians engaging in the correct identification of CELs. The model is designed to work with conventional clinical MRI images. To ensure clinical applicability, we developed the model utilizing clinical MRI scans collected as part of the Swiss Multiple Sclerosis Cohort (SMSC) study [23], in a multicentric dataset including multiple time points both with and without CELs. A specific objective of the model was to ensure the segmentation of lesions with a small volume (as low as three voxels, equivalent to 3 mm^3^), a challenging aspect in clinical settings requiring manual intervention.

## 2. Materials and Methods

### 2.1. Dataset

The dataset was collected using 1.5- and 3-Tesla MRI systems across 7 centers affiliated with the SMSC study [23]. MRI acquisitions included T1-weighted (T1w) magnetization prepared rapid gradient echo (MPRAGE) before and after the administration of gadolinium, and FLAIR images. MRI acquisition protocols are detailed in Appendix A, Table A1.

The automatic detection and segmentation of WML were conducted with a deep learning-based approach [24], followed by manual correction. The resulting WML masks were then incorporated into the input dataset. CELs were manually segmented by 2 experts (one neurologist and one radiologist) by consensus. The dataset encompassed 372 scans from 280 patients with MS, categorized into scans with and without CELs (Table 1). The inclusion of patients without CELs was motivated by the aim of creating a method ensuring good performance in real-world clinical scenarios, where only a minority of patients exhibit CELs [25].

The demographic and clinical characteristics of the cohort are described in Table 2.

Sixty-two patients underwent more than one MRI scan. Each scan from multiple visits of a patient was treated independently and incorporated into the same dataset for training, validation, or testing.

The different MRI contrasts from the same time point were co-registered using the Elastix toolbox 4.9 [26] and resampled to 1 × 1 × 1 mm^3^.

Skull-stripping was performed using HD-BET [27] to eliminate non-brain tissue.

### 2.2. Preprocessing and Sampling Strategy

The model’s input comprised patches extracted from preprocessed images to which data augmentation [28] was applied to mitigate overfitting [29]. Preprocessing steps included the image intensity z-score normalization and cropping of 32 patches (64 × 64 × 64 mm^3^) from each image. The cropping strategy varied based on the presence of CELs in the scans.

- For scans with CELs: 32 patches of 64 × 64 × 64 mm^3^ were randomly cropped, with one out of every three patches centered on CELs (positive patches) and the remaining two centered on WMLs (negative patches).
- For scans without CELs: 32 patches of 64 × 64 × 64 mm^3^ were randomly cropped, all centered on WMLs.

Subsequently, the obtained patches underwent a second cropping with a random center, resulting in a final size of 48 × 48 × 48 mm^3^. Additionally, patches went through random flipping, rotation by 90 degrees, and intensity shifting by 0.25. Additionally, a random affine transformation was applied to the patches.

To maintain consistency between the training and the validation process to ensure a more reliable performance assessment and better generalization to new data, WML sampling was used during validation after intensity normalization, replacing sliding window inference. Sliding window inference involves cropping patches across the entire image, presenting a distinct technique compared to the training process. To maintain alignment with the training sampling strategy, patches were cropped specifically from areas where WMLs were detected and subsequently utilized as input for the model inference. 

**Figure 1:**
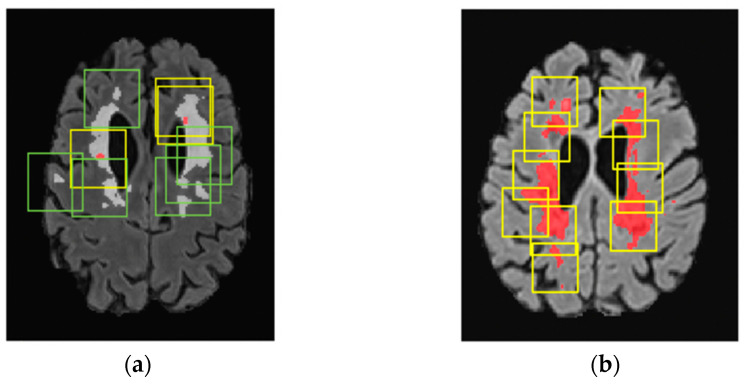
Sampling strategy reported in T1-w images with gadolinium-contrast agent. (**a**) Sampling strategy in the training process. One-third of the cubes (yellow) are centered in CELs (segmented in red), and two-thirds (green) are centered in WMLs (segmented in white). (**b**) Sampling strategy in the validation process where all regions with WMLs (in red) were sampled.

The predicted patches were mapped back from where they were extracted so that we could compute the DSC in the whole image. Overlapping voxel predictions were summed, and values exceeding 1 were converted to 1, resulting in a fully reconstructed prediction image. Hence, the validation strategy paralleled the training one, with all potential regions where CELs might be present given as input to the model (refer to Figure 1).

### 2.3. Network Architecture

In this work, a 3D patch-based U-Net-based network originally developed for the segmentation of cortical lesions [24] composed of (32, 64, 64, 128, 128) convolutional filters in the encoder and (256, 128, 128, 64, 64, 1) filters in the decoder was expanded. This strategy was motivated by the presence of similarities between cortical lesions and CELs in terms of rarity and small volume. The proposed neural network consists of 5 convolutional layers of (32, 64, 128, 256, 512) filters in the encoder and (512, 256, 128, 64, 32) in the decoder. The stride parameter is settled to two, and the activation function is PReLu. The neural network was implemented in Python 3.8.5 using the MONAI library 0.5.2 [30].

### 2.4. Metrics

The True Positive Rate (TPR) and False Positive Rate (FPR), defined as follows, were utilized to evaluate the detection performance of the model.

(1) $$True\;positive\;rate=\frac{n\;\;true\;positive\;lesions}{n\;true\;positive\;lesions+n\;false\;negative\;lesions}$$

(2) $$False\;positive\;rate=\frac{n\;false\;positive\;lesions}{n\;true\;positive\;lesions+n\;false\;positive\;lesions}$$

where *n* True Positive lesions, *n* False Positive lesions, and *n* False Negative lesions are the number of True Positive, False Positive, and False Negative lesions, respectively.

The Dice Score Coefficient was utilized to measure the segmentation performance of the network, and it measures the voxel similarity between the ground truth and the model output mask.

(3) $$Dice\;score\;coefficient=\frac{2\times n\;true\;positive\;voxels}{2\times n\;true\;positive\;voxels+n\;false\;negative\;voxels+n\;false\;positive\;voxels}$$

The best model was applied in the training both with the starting loss function and with the proposed weighted loss function.

The DSC was calculated both as the mean value across all patches and as the mean value across the entire images.

The patches were considered only if a TP lesion was present and lesions that did not overlap with the WML masks were deleted. During this procedure, we did not apply the minimum threshold of 3 voxels, because the patch could include only a portion of CELs.

These portions, when combined with other patches, contribute to forming the entire lesion.

For the whole images, lesions outside the WML and lesions with a volume lower than 3 voxels were deleted. The Dice Score Coefficient in whole images was calculated only for images with at least one TP lesion.

To mitigate potential ambiguity arising from scans lacking CELs, the Dice Score Coefficient was individually calculated for each True Positive CEL. Furthermore, the DSC was stratified based on the ground truth lesion dimensions, enabling the assessment of the network’s performance across various lesion volume categories.

### 2.5. Training Pipeline

The loss function in La Rosa et al., 2022 [31,32], starting loss function, was a linear combination of the dice loss function and the focal loss function with a gamma parameter of 2.

(4) $$Loss_{function}=0.5\times \left(Loss_{Dice}\right)+Loss_{focal}$$

The focal loss function [33] can be considered a variation in the cross-entropy loss [34] that works well for high-imbalance datasets by emphasizing more challenging samples. Meanwhile, the dice loss function [35] is employed to assess the similarity between the two binary images. Considering the dataset’s imbalance, there is a higher count of negative patches compared to positive ones (rate_unbalance_ = $\frac{2}{9}$).

An important point to emphasize is that the dice loss attains a value of 1 within negative patches when there is a nonzero count of False Positive (FP) voxels. As a consequence, the learning process gives low importance to the decrease in the dice loss in the positive patches (Loss_Dice_positive_), but concentrates more on lowering in the dice loss in the negative patches (Loss_Dice_negative_). Therefore, we defined a new loss function to reduce the weight of Loss_Dice_negative_, multiplying it with the rate of patches’ imbalance to focus more on the segmentation of CELs in the positive patches:

(5) $$Loss_{function}=mean\left(rate_{imbalance}*Loss_{Dice_{negative}}+Loss_{Dice_{positive}}\right)+mean\left(Loss_{focal_{negative}}+Loss_{focal_{positive}}\right)$$

The trends of the training and validation loss function during the training phase are reported in the Supplementary Materials, Figure S1.

Adam with an initial learning rate of 0.00005 was used as an optimizer [36].

Cross-validation was carried out by randomly dividing the multicenter dataset into 11 subgroups at the patient level. One subgroup was defined as a test dataset, and at each iteration, one subgroup was designated as the validation set while the remaining 9 subgroups were employed for training purposes.

**Figure 2:**
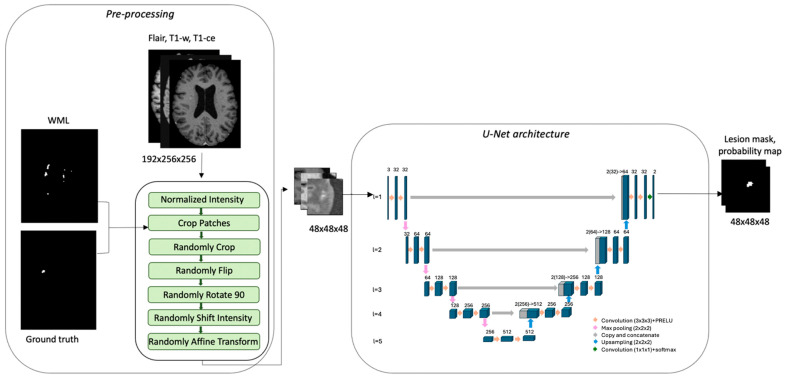
Training process for assessment with CELs. FLAIR, T1-weighted, and T1-weighted with contrast agent are the input dataset. The pre-processing pipeline crops the patches using WMLs and ground truth masks. The patches are the input of the U-Net model that creates the probability and CEL mask.

An example of the training pipeline is reported in Figure 2.

To determine the optimal model, the Dice Score Coefficient was computed over the entire image during the validation process, and the highest value was employed.

The model’s predictions were filtered to exclude output lesions situated beyond the WML mask. If the predicted CEL overlapped less than 10% of the WML, it was excluded.

## 3. Results

### 3.1. Comparative Analysis of Loss Functions

The results for each category are reported in Table 3. The weighted loss function exhibited higher performance both in the validation and test datasets. Indeed, the weighted loss function increased the weights of Loss_Dice_positive_, thereby improving the DSC in positive patches. The DSC in whole images was also higher.

### 3.2. Model Performance

Table 4 shows that the highest concentration of incorrect detections occurred for low-volume CELs, with the highest number of False Negative lesions displaying a volume lower than 10 mm^3^. In terms of the overall segmentation performance, although the DSC varied across all lesion volume groups, the variation was not substantial. The average DSC for all the True Positive (TP) lesions was 0.76. The model resulted in a True Positive Rate and a False Positive Rate of 0.93 and 0.02.

## 4. Discussion

In this study, we developed a deep learning model to automatically detect and segment CELs in clinical MRI images of people with MS. The model achieved a True Positive Rate of 0.93 and a False Positive Rate of 0.02, rendering this method an attractive clinical decision support tool in the clinical care of MS patients.

In the initial phase, we constructed a UNet-based network integrating a loss function that comprised a linear combination of focal and dice loss. We introduced the sampling strategy which played a crucial role in reducing FP predictions and achieving stable performance metrics, such as the DSC and loss function, during the training process.

Furthermore, we modified the loss function to account for the dataset imbalance. This adjustment was made to reduce the impact of the dice loss on negative patches, i.e., patches sampled on non-CELs, given that negative patches are more numerous than positive ones, i.e., patches sampled on CELs. The purpose of this change is to better accommodate the specific characteristics of our dataset. The weighted loss function employed in this study contributed to high performance on validation and test datasets.

The dataset we used to validate this method belongs to the SMSC and, after the preprocessing stage, exhibits a high imbalance between patches containing CELs and those without. The input images for our tool included T1-weighted, T1-weighted with gadolinium contrast, FLAIR, and the WML mask. A few other published methods for CEL detection/segmentation did not require WML masks; however, the tools developed by Coronado et al. [17] and Gaj et al. [16] required additional MRI contrasts (T2-weighted and Proton Density-weighted) compared to the dataset in this study.

In this study, the model operated with an imbalanced dataset, mimicking real clinical scenarios—a methodology that was also followed by Krishnan et al. [18] and Coronado et al. [17]—in order to enhance the model’s ability to generalize and effectively distinguish between the two classes. 

However, unlike what we did, Gaj et al. [16] trained the model only on patients exhibiting at least one CEL and assessed the model performance in patients without CELs only in the test dataset.

The objective of our work was to create a deep learning model intended for integration into clinical practice. Consequently, the dataset utilized in this study comprised routine clinical MRI data, similar to the datasets used in the studies conducted by Gaj et al. [16] and Schlaeger et al. [19]. In contrast, the studies conducted by Krishnan et al. [18] and Coronado et al. [17] incorporated datasets from clinical trials.

Overcoming some of the limitations of previous studies, our method ensures the detection of small CELs, which is crucial in clinical practice, both for diagnostic procedures and therapeutic follow-up. Notably, the network proposed in this study considered lower-volume lesions (3 mm^3^) compared to other investigations (2.5–32.5, 20, 9 mm^3^) [16,17,18].

However, it is important to acknowledge that we used a cohort for training and validation (340 MRI scans) that was smaller than the one used in some previous studies (Coronado et al. [17], Krishnan et al. [18], and Schlaeger et al. [19]—805, 2971, 1488 MRI scans), although our work yielded comparable results.

In terms of segmentation performance, the Dice Score Coefficient achieved in our study (0.76) was higher compared to Gaj et al. [16] (0.698) and comparable to those obtained by Coronado et al. [17] (0.77) and Krishnan et al. [18] (0.77). Yet, our study demonstrated improved detection performance in terms of the True Positive Rate and False Positive Rate (0.93/0.02) compared to Gaj et al. [16] (0.844, 0.307), Krishnan et al. [18] (0.88, 0.04), and Coronado et al. [17] (0.90, 0.23); Schlaeger et al. [19] report 73 True Positive lesions, 91 False Negative lesions, and 22 False Positive lesions (True Positive Rate of 0.44 and False Positive Rate of 0.23).

**Figure 3:**
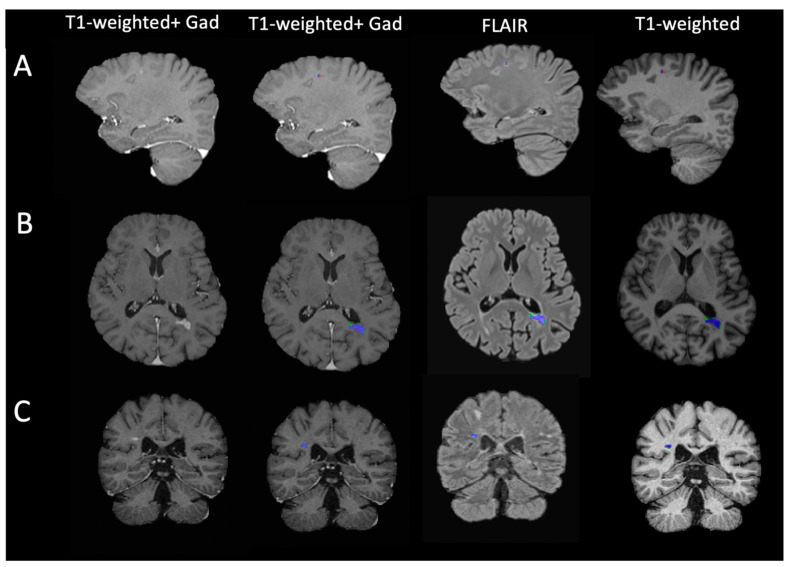
Model lesion segmentation mask and MRI input images. Each row reports distinct patients (**A**–**C**) exhibiting varied lesions characterized by differences in shape, volume, and spatial positioning. The blue areas represent voxels classified as True Positives, the green areas denote False Positive voxels, and the red regions indicate False Negative detections.

When discussing the performance achieved in different studies, it is crucial to note that CELs typically exhibit low volumes [12]. Consequently, a low number of voxel variations between the ground truth mask and the prediction leads to a high decrease in the Dice Score Coefficient. Figure 3 illustrates three examples of predicted lesion masks compared to the ground truth when applied to the input images. The discrepancy between the two masks, delineated by green for False Positive voxels and red for False Negative voxels, primarily occurs at the lesion border.

One limitation of this study is the relatively small size of the dataset. To address this challenge, we augmented the input dataset to provide outcomes that are more generalizable and potentially diminish the count of False Negative lesions. In future work, we will consider applying the tool to a larger dataset.

Additionally, a limitation arises from incorporating WML masks in the input dataset, necessitating both automated and subsequent manual correction. These masks are typically integrated into the datasets due to their clinical relevance, encompassing clinical information. Therefore, the necessity of these masks should not pose a constraint in most scenarios. However, their usage in the sampling methodology relies on the segmentation of WML masks. In the future, it will be interesting to evaluate the model’s performance using only fully automated lesion masks.

To further improve model performance, self-supervised learning [37] can be employed to address the limitations posed by the small amount of manually segmented data. Additionally, integrating feature-preserving mesh networks [38] could help identify and preserve structural features of contrast-enhanced lesions (CELs).

## 5. Conclusions

In conclusion, the strategies implemented in our UNet-based network permit the detection and segmentation of low-volume and sparse CELs in MRI images. 

The procedure has the potential to assist clinicians in the identification of CELs in clinical practice. This procedure holds fundamental importance in clinical practice because it enables the diagnosis and monitoring of treatment in patients with multiple sclerosis.

Remarkably, we showed a good performance of the tool in a real-world multicentric clinical scenario. The sampling strategy and the weighted loss function were introduced to overcome the scarcity and the heterogeneity of CELs.

Consequently, the model demonstrated comparable segmentation performance and exhibited enhanced identification capabilities when compared to previous studies. These results support the network’s potential suitability for clinical application.

## Figures and Tables

**Table 1:** Composition of the dataset in terms of number of scans and patients with and without contrast-enhancing lesions (CELs).

	Number of Patients with MS	Number of MRI Scans	Number of CELs	Mean Number of CELs per Scan	Mean Volume of CELs (mm^3^)
Data with CELs	162	208	654	3	136
Data without CELs(control cases)	118	164	0	0	-
Total	280	372	654	1.7	

**Table 2:** Demographic and clinical characteristics of the cohort.

Demographic and Clinical Data	*n* = 372 MRI Scans
Female, No. (%)	264 (71)
Male, No. (%)	108 (29)
Age at closest visit, mean (SD), y	41.6 (11.5)
Disease duration at closest visit, mean (SD), y	11.8 (9.4)
EDSS at closest visit, median (IQR)	2.0 (1.5, 3.5)
CIS, No. (%)	7 (1.9)
PPMS, No. (%)	7 (1.9)
RRMS, No. (%)	336 (91.1)
SPMS, No. (%)	19 (5.1)

Abbreviations: EDSS, Expanded Disability Status Scale; CIS, clinically isolated syndrome; PPMS, Primary Progressive Multiple Sclerosis; RRMS, Relapsing-Remitting Multiple Sclerosis; SPMS, Secondary Progressive Multiple Sclerosis.

**Table 3:** Comparison of the best model, defined by the highest DSC in the whole image, using the starting and the weighted loss function. The metrics are calculated using the model predictions for both the validation dataset and test dataset, which is highlighted in blue.

Dataset	Loss Function	DSC in Patches	DSC in Whole Images	Number of TP Lesions	Number of FN Lesions	Number of FP Lesions
Validation	Starting	0.78	0.80	39	8	5
Validation	Weighted	0.78	0.82	40	7	6
Test	Starting	0.67	0.72	50	10	3
Test	Weighted	0.72	0.76	56	4	1

**Table 4:** Performance of the model in the test dataset subdivided by lesion volume. The DSC coefficient is the mean of the DSC for each lesion in the volume range. Regarding TP and FN lesions, the subdivision was performed considering the ground truth lesion volume. On the other hand, the FPs are classified based on their volume.

Lesion Volume (mm^3^)	Number of TP Lesions	Number of FN Lesions	Number of FP Lesions	Dice Score Coefficient
3–10	6	3	1	0.88
10–20	5	1	0	0.56
20–30	6	0	0	0.73
30–40	4	0	0	0.82
40–50	3	0	0	0.61
50–100	16	0	0	0.79
100–200	11	0	0	0.82
200–300	1	0	0	0.85
>300	4	0	0	0.83
All	56	4	1	0.76

## Data Availability

The data presented in this study are available on request from the Swiss MS Cohort.

## Supplementary Materials

The following supporting information can be downloaded at https://www.mdpi.com/article/10.3390/bioengineering11080858/s1, Figure S1: Training and validation loss functions throughout the training process.

## Appendix A

Swiss Multiple Sclerosis Magnetic Resonance Imagining protocols for the dataset.

**Table A1:** Swiss Multiple Sclerosis (SMSC) Magnetic Resonance Imaging (MRI) protocols for the dataset.

Center	MRI Scanner Model	Number of Scans	Sequence	Resolution (mm^3^)	Flip Angle (degree)	Repetition Time (ms)	Echo Time (ms)	Inversion Time (ms)
			FLAIR	1 × 1 × 1	120	5000	398	1800
Lausanne	SIEMENS Skyra 3T	51	T1n	1 × 1 × 1.2	9	2300	2.9	900
			T1ce	1 × 1 × 1	9	2000	2.03	1100
			FLAIR	1 × 1 × 1	120	5000	395	1600
Geneva	SIEMENS Skyra 3T	16	T1n	1 × 1 × 1	9	2000	2.03	1100
			T1ce	1 × 1 × 1	9	2000	2.03	1100
			FLAIR	1 × 1 × 1	120	5000	335	1800
Bern	SIEMENS Skyra 3T	26	T1n	1 × 1 × 1	15	1790	2.58	1100
			T1ce	1 × 1 × 1	15	2060	53	1100
			FLAIR	1 × 1 × 1	120	5000	280	1600
Basel	SIEMENS Skyra 3T	245	T1n	1 × 1 × 1	9	2300	1.96	900
			T1ce	1 × 1 × 3	30	30	11	0
			FLAIR	1 × 1 × 1.3	120	7500	317	3000
Aarau	SIEMENS Skyra 3T	14	T1n	1 × 1 × 1	15	1970	3.14	1100
			T1ce	1 × 1 × 1	9	2100	4.78	1800
			FLAIR	1 × 1 × 1	120	5000	373	900
Lugano	SIEMENS Skyra 3T	7	T1n	1 × 1 × 1	9	2300	2.98	950
			T1ce	1 × 1 × 1	120	600	11	0
			FLAIR	1 × 1 × 1	120	6000	355	1850
St. Gallen	SIEMENS Avanto 1.5T	13	T1n	1 × 1 × 1	8	2700	2.96	950
			T1ce	1 × 1 × 1	8	2700	2.96	950

FLAIR: FLuid Attenuated Inversion Recovery; T1n: T1-weighted; T1ce: T1-weighted with gadolinium contrast agent.

## References

[1] Lassmann H., van Horssen J., Mahad D. (2012). Progressive multiple sclerosis: Pathology and pathogenesis. Nat. Rev. Neurol..

[2] Brück W. (2005). The pathology of multiple sclerosis is the result of focal inflammatory demyelination with axonal damage. J. Neurol..

[3] Miller D.H., Barkhof F., Nauta J.J.P. (1993). Gadolinium enhancement increases the sensitivity of MRI in detecting disease activity in multiple sclerosis. Brain.

[4] Granziera C., Reich D.S. (2020). Gadolinium should always be used to assess disease activity in MS—Yes. Mult. Scler. J..

[5] Montalban X., Gold R., Thompson A.J., Otero-Romero S., Amato M.P., Chandraratna D., Clanet M., Comi G., Derfuss T., Fazekas F. (2018). ECTRIMS/EAN Guideline on the pharmacological treatment of people with multiple sclerosis. Mult. Scler. J..

[6] Kira J.-I. (2021). Redefining use of MRI for patients with multiple sclerosis. Lancet Neurol..

[7] Guo B.J., Yang Z.L., Zhang L.J. (2018). Gadolinium Deposition in Brain: Current Scientific Evidence and Future Perspectives. Front. Mol. Neurosci..

[8] Tsantes E., Curti E., Ganazzoli C., Puci F., Bazzurri V., Fiore A., Crisi G., Granella F. (2020). The contribution of enhancing lesions in monitoring multiple sclerosis treatment: Is gadolinium always necessary?. J. Neurol..

[9] Wattjes M.P., Ciccarelli O., Reich D.S., Banwell B., de Stefano N., Enzinger C., Fazekas F., Filippi M., Frederiksen J., Gasperini C. (2021). 2021 MAGNIMS–CMSC–NAIMS consensus recommendations on the use of MRI in patients with multiple sclerosis. Lancet Neurol..

[10] Lesjak Ž., Galimzianova A., Koren A., Lukin M., Pernuš F., Likar B., Špiclin Ž. (2018). A Novel Public MR Image Dataset of Multiple Sclerosis Patients With Lesion Segmentations Based on Multi-rater Consensus. Neuroinformatics.

[11] Mortazavi D., Kouzani A.Z., Soltanian-Zadeh H. (2012). Segmentation of multiple sclerosis lesions in MR images: A review. Neuroradiology.

[12] Filippi M., Preziosa P., Banwell B.L., Barkhof F., Ciccarelli O., De Stefano N., Geurts J.J.G., Paul F., Reich D.S., Toosy A.T. (2019). Assessment of lesions on magnetic resonance imaging in multiple sclerosis: Practical guidelines. Brain.

[13] Danieli L., Roccatagliata L., Distefano D., Prodi E., Riccitelli G., Diociasi A., Carmisciano L., Cianfoni A., Bartalena T., Kaelin-Lang A. (2022). Nonlesional Sources of Contrast Enhancement on Postgadolinium “Black-Blood” 3D T1-SPACE Images in Patients with Multiple Sclerosis. Am. J. Neuroradiol..

[14] Lundervold A.S., Lundervold A. (2019). An overview of deep learning in medical imaging focusing on MRI. Z. Fur Med. Phys..

[15] Kayalibay B., Jensen G., van der Smagt P. (2017). CNN-based Segmentation of Medical Imaging Data. arXiv.

[16] Gaj S., Ontaneda D., Nakamura K. (2021). Automatic segmentation of gadolinium-enhancing lesions in multiple sclerosis using deep learning from clinical MRI. PLoS ONE.

[17] Coronado I., Gabr R.E., Narayana P.A. (2020). Deep learning segmentation of gadolinium-enhancing lesions in multiple sclerosis. Mult. Scler. J..

[18] Krishnan A.P., Song Z., Clayton D., Gaetano L., Jia X., de Crespigny A., Bengtsson T., Carano R.A.D. (2022). Joint MRI T1 Unenhancing and Contrast-enhancing Multiple Sclerosis Lesion Segmentation with Deep Learning in OPERA Trials. Radiology.

[19] Schlaeger S., Shit S., Eichinger P., Hamann M., Opfer R., Krüger J., Dieckmeyer M., Schön S., Mühlau M., Zimmer C. (2023). AI-based detection of contrast-enhancing MRI lesions in patients with multiple sclerosis. Insights Into Imaging.

[20] Weiss K., Khoshgoftaar T.M., Wang D.D. (2016). A survey of transfer learning. J. Big Data.

[21] Wahlig S.G., Nedelec P., Weiss D.A., Rudie J.D., Sugrue L.P., Rauschecker A.M. (2023). 3D U-Net for automated detection of multiple sclerosis lesions: Utility of transfer learning from other pathologies. Front. Neurosci..

[22] Huang L., Zhao Z., An L., Gong Y., Wang Y., Yang Q., Wang Z., Hu G., Wang Y., Guo C. (2024). 2.5D transfer deep learning model for segmentation of contrast-enhancing lesions on brain magnetic resonance imaging of multiple sclerosis and neuromyelitis optica spectrum disorder. Quant. Imaging Med. Surg..

[23] Disanto G., Benkert P., Lorscheider J., Mueller S., Vehoff J., Zecca C., Ramseier S., Achtnichts L., Findling O., Nedeltchev K. (2016). The Swiss Multiple Sclerosis Cohort-Study (SMSC): A Prospective Swiss Wide Investigation of Key Phases in Disease Evolution and New Treatment Options. PLoS ONE.

[24] La Rosa F., Abdulkadir A., Fartaria M.J., Rahmanzadeh R., Lu P.-J., Galbusera R., Barakovic M., Thiran J.-P., Granziera C., Cuadra M.B. (2020). Multiple sclerosis cortical and WM lesion segmentation at 3T MRI: A deep learning method based on FLAIR and MP2RAGE. NeuroImage Clin..

[25] Khaleeli Z., Ciccarelli O., Mizskiel K., Altmann D., Miller D., Thompson A. (2010). Lesion enhancement diminishes with time in primary progressive multiple sclerosis. Mult. Scler. J..

[26] Klein S., Staring M., Murphy K., Viergever M.A., Pluim J.P.W. (2009). elastix: A Toolbox for Intensity-Based Medical Image Registration. IEEE Trans. Med. Imaging.

[27] Isensee F., Schell M., Pflueger I., Brugnara G., Bonekamp D., Neuberger U., Wick A., Schlemmer H.-P., Heiland S., Wick W. (2019). Automated brain extraction of multisequence MRI using artificial neural networks. Hum. Brain Mapp..

[28] Chlap P., Min H., Vandenberg N., Dowling J., Holloway L., Haworth A. (2021). A review of medical image data augmentation techniques for deep learning applications. J. Med. Imaging Radiat. Oncol..

[29] Rice L., Wong E., Kolter J.Z. (2020). Overfitting in Adversarially Robust Deep Learning. https://github.com/.

[30] Cardoso M.J., Li W., Brown R., Ma N., Kerfoot E., Wang Y., Murrey B., Myronenko A., Zhao C., Yang D. (2022). MONAI: An open-source framework for deep learning in healthcare. arXiv.

[31] La Rosa F., Beck E.S., Maranzano J., Todea R., van Gelderen P., de Zwart J.A., Luciano N.J., Duyn J.H., Thiran J., Granziera C. (2022). Multiple sclerosis cortical lesion detection with deep learning at ultra-high-field MRI. NMR Biomed..

[32] (2019). ECTRIMS 2019—Poster Session 1. Mult. Scler. J..

[33] Lin T.Y., Goyal P., Girshick R., He K., Dollar P. (2020). Focal loss for dense object detection. IEEE Trans. Pattern Anal. Mach. Intell..

[34] Ma Y.-d., Liu Q., Qian Z.-B. Automated Image Segmentation Using Improved PCNN Model Based on Cross-entropy. Proceedings of the 2004 International Symposium on Intelligent Multimedia, Video and Speech Processing.

[35] Sudre C.H., Li W., Vercauteren T., Ourselin S., Cardoso M.J. (2017). Generalised dice overlap as a deep learning loss function for highly unbalanced segmentations. Lecture Notes in Computer Science (Including Subseries Lecture Notes in Artificial Intelligence and Lecture Notes in Bioinformatics).

[36] Kingma D.P., Ba J. (2014). Adam: A Method for Stochastic Optimization. arXiv.

[37] Krishnan R., Rajpurkar P., Topol E.J. (2022). Self-supervised learning in medicine and healthcare. Nat. Biomed. Eng..

[38] Imran S.M.A., Saleem M.W., Hameed M.T., Hussain A., Naqvi R.A., Lee S.W. (2023). Feature preserving mesh network for semantic segmentation of retinal vasculature to support ophthalmic disease analysis. Front. Med..

