# Supplementary material for: Contrast-Enhancing Lesion Segmentation in Multiple Sclerosis: A Deep Learning Approach Validated in a Multicentric Cohort
Source: Bioengineering (Basel). 2024 Aug 22;11(8):858. doi: 10.3390/bioengineering11080858 (PMC11351944; doi:10.3390/bioengineering11080858)
Supplement: Supplementary file 1 [file bioengineering-11-00858-s001.zip › bioengineering-3127940-supplementary.pdf]

bioengineering

Supplementary Materials

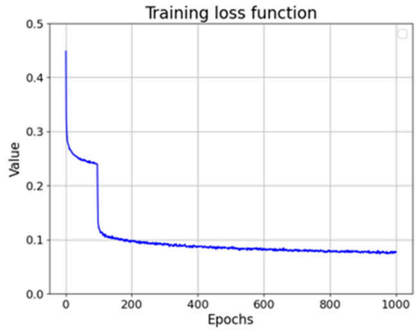

(**a**)

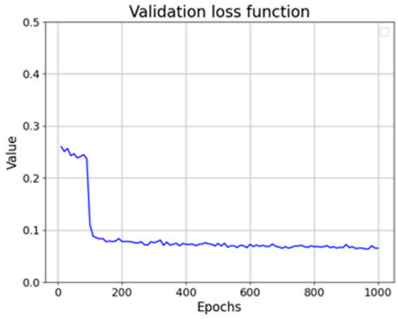

(**b**)

Figure S1. Loss functions evolution during the training process: (**a**) Training loss function; (**b**) Validation loss function.
